# Relative impacts of environmental variation and evolutionary history on the nestedness and modularity of tree–herbivore networks

**DOI:** 10.1002/ece3.1559

**Published:** 2015-07-03

**Authors:** Kathryn M Robinson, Céline Hauzy, Nicolas Loeuille, Benedicte R Albrectsen

**Affiliations:** 1 Department of Forest Genetics and Plant Physiology, Umeå Plant Science Centre, Swedish University of Agricultural Sciences901 83, Umeå, Sweden; 2 Department of Plant Physiology, Umeå Plant Science Centre, Umeå University901 87, Umeå, Sweden; 3 Institute of Ecology and Environmental Sciences of Paris, UMR7618, UPMC-CNRS7 quai St Bernard, 75005, Paris, France; 4 Department of Plant and Environmental Sciences, University of CopenhagenThorvaldsensvej 40, DK 1871, Frederiksberg C, Denmark

**Keywords:** Antagonism, arthropod, aspen, bipartite networks, degree of specialization, modularity, nestedness, trophic strength

## Abstract

Nestedness and modularity are measures of ecological networks whose causative effects are little understood. We analyzed antagonistic plant–herbivore bipartite networks using common gardens in two contrasting environments comprised of aspen trees with differing evolutionary histories of defence against herbivores. These networks were tightly connected owing to a high level of specialization of arthropod herbivores that spend a large proportion of the life cycle on aspen. The gardens were separated by ten degrees of latitude with resultant differences in abiotic conditions. We evaluated network metrics and reported similar connectance between gardens but greater numbers of links per species in the northern common garden. Interaction matrices revealed clear nestedness, indicating subsetting of the bipartite interactions into specialist divisions, in both the environmental and evolutionary aspen groups, although nestedness values were only significant in the northern garden. Variation in plant vulnerability, measured as the frequency of herbivore specialization in the aspen population, was significantly partitioned by environment (common garden) but not by evolutionary origin of the aspens. Significant values of modularity were observed in all network matrices. Trait-matching indicated that growth traits, leaf morphology, and phenolic metabolites affected modular structure in both the garden and evolutionary groups, whereas extra-floral nectaries had little influence. Further examination of module configuration revealed that plant vulnerability explained considerable variance in web structure. The contrasting conditions between the two gardens resulted in bottom-up effects of the environment, which most strongly influenced the overall network architecture, however, the aspen groups with dissimilar evolutionary history also showed contrasting degrees of nestedness and modularity. Our research therefore shows that, while evolution does affect the structure of aspen–herbivore bipartite networks, the role of environmental variations is a dominant constraint.

## Introduction

Understanding the organization of ecological networks is a key issue in community and functional ecology. Early models, explicitly compared to different data sets, clearly suggest that network architecture differs from random (Cohen et al. 1990; Williams and Martinez 2000), food webs being “small world” systems in which any two species are linked by short paths (Montoya and Solé 2002) and interact with constrained subsets of the total network (Krause et al. 2003; Montoya et al. 2006). Several recent studies (i.e., Bascompte et al. 2006; Fontaine et al. 2011) particularly tackle the structure of bipartite networks (i.e., networks with two groups of species of different types, such as plant–herbivore, plant–pollinator, or host–parasite networks). Such networks show contrasting levels of modularity and nestedness. Modularity represents the propensity of the network to exhibit clusters of species that interact more strongly together than with the rest of the network (Krause et al. 2003), while nestedness measures the degree to which interactions of specialists are a subset of interactions of generalists (Bascompte et al. 2003). Fundamental questions arise regarding mechanisms that can explain such network architecture. Furthermore, the increasing recognition that modularity and nestedness are intimately linked to network dynamics and robustness (Thébault and Fontaine 2010) implies that their consequences for the management and conservation of species may be far-reaching. They therefore have an applied value for management and conservation of ecosystem services and species diversity.

Patterns of nestedness and modularity exhibit systematic variation among systems. In general, networks may be characterized by the dominant type of interaction they represent. Thus, mutualistic networks (e.g., plant–pollinator networks) tend to be more nested and antagonistic networks (e.g., plant–herbivore networks) more modular (Fontaine et al. 2011). Modularity and nestedness are also usually negatively correlated (Fontaine et al. 2011). However, within each type of network, structures also vary. While antagonistic networks usually have lower nestedness, it has been proposed that “intimate” antagonistic networks (in the sense that the consumer is highly specialized and spends most of its life cycle on its host) are more modular and less nested than promiscuous or more loosely tied antagonistic networks (Van Veen et al. 2008). Interestingly, this relationship is also true in mutualistic networks, where more intimate interactions (Ollerton et al. 2003) seem to lead to lower levels of nestedness (Guimarães et al. 2007; Thompson et al. 2013).

Systematic variations in the architecture of ecological networks, which may be assigned to the dominant interaction type and the degree of intimacy that the partners display, highlight the potential that network patterns, such as nestedness and modularity, may be explained by general behavioral and evolutionary mechanisms. Different hypotheses have been proposed in this regard, for example, stating that these structures are indicative of community stability (Thébault and Fontaine 2010) and may describe community processes such as competitive exclusion (Bastolla et al. 2009). Both modularity and nestedness heavily depend on the degree of specialization within the bipartite network. Therefore, any adaptation that involves trait-matching, diet breadth, or prey vulnerability may also affect modularity or nestedness. Theoretical models illustrate different ways through which the evolutionary dynamics of such traits may affect network architecture. In mutualistic networks, selection pressure that shapes the coevolution of mutual dependencies of plants and animals on the partner species leads to nested structures (Bascompte et al. 2006); in antagonistic networks, evolution also greatly impacts the network architecture. Evolution of plant defences, for instance, leads to modular, lowly connected food webs in rich patches or when dispersal is high along environmental gradients, while such modularity disappears in less extreme scenarios (Loeuille and Leibold 2008). Adaptive foraging associated with body size coevolution between prey and predators may create modular networks, provided the consumer diet breadth is heavily constrained (Loeuille and Loreau 2005, 2009). Trait variation, however, not only arises through evolutionary dynamics, but also due to environmental filtering acting on a regional species pool. Environmental conditions per se therefore likely explain part of the modularity or nestedness of interaction webs, and climatic factors, for example, influence the architecture of pollination networks (Dalsgaard et al. 2013).

As all of these different mechanisms can explain variations in nestedness and modularity, a crucial next step is to understand their relative importance. Studying multiple marine mutualistic goby–shrimp networks, Thompson et al. (2013) showed that nestedness is best explained by habitat use (measured from different abiotic and physical parameters) and phylogenetic history (measured from phylogenetic dissimilarity matrices). This suggests that the interplay of evolution and local ecological dynamics is instrumental in shaping the architecture of this system. In this work, we tackle the very same question, that is, the relative importance of environmental constraints and evolutionary history of the network architecture. Network analyses have so far been restricted to interspecific webs. Long-lived keystone species such as aspen show strong variation in defence-related traits and are obvious candidates for increasing our understanding of bipartite antagonistic networks based on intraspecific phenotypic variations.

**Figure 1:**
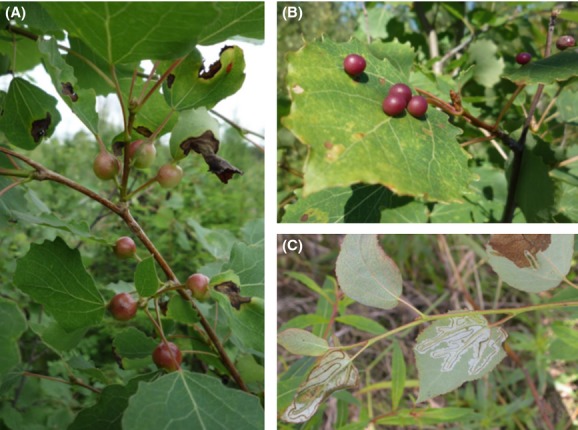
Aspen canopies are inhabited by a community of mainly specialist arthropod herbivores. The galling midges (A) *Contarinia petioli* (morphospecies no. 9) and (B) *Harmandia tremulae* (morphospecies no. 18) leave distinctive features in the foliage of young trees and may be identified to the species level. Serpentine mines (C) are made by the lepidopteran *Phyllocnistis labyrinthella* (morphospecies no. 28) and trenching chewers (C) like the sluglike larvae of the sawfly *Caliroa tremulae* (morphospecies no. 5) also leave characteristic marks on aspen leaves.

**Figure 2:**
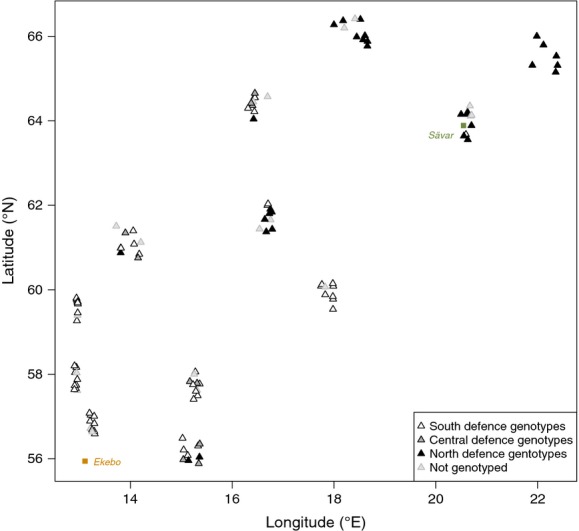
Latitudes and longitudes within Sweden indicating the location of the two common gardens (colored squares), Ekebo (South garden) and Sävar (North garden), and the origins of the genotypes (triangles) comprising the SwAsp collection that were cloned and planted in the gardens, shaded according to their respective defence genotype cluster (DGC). Raw data are available (Robinson et al. 2015).

We study the architecture of herbivory networks involving different aspen (*Populus tremula*) genotypes and associated arthropod herbivores (Fig.1). This system has several advantages that we aim to exploit. Firstly, we compare the networks based on the Swedish Aspen (SwAsp) collection, a set of wild aspen genotypes grown in two common gardens (Fig.2) that differ in terms of abiotic and energetic conditions, resulting in contrasting tree biomass (Table1). Secondly, we rely on a detailed knowledge of the evolutionary history of poplar genotypes. Previous work analyzing single nucleotide polymorphisms (SNPs) in seven genes conferring defence against chewing insects identified a division of the SwAsp collection into two distinct groups of genotypes, one predominantly from southern and one from northern Sweden (Bernhardsson and Ingvarsson 2012), which we refer to as the Northern and Southern defence genotype cluster (South/North DGC, respectively). The geographic distribution of these genotype clusters was found to mirror that of arthropod herbivore abundance data and the untargeted metabolome in common garden data (Bernhardsson et al. 2013). Genetic association mapping has identified correlations between the selected SNPs and arthropod herbivore abundances including chewing insects and several feeding guilds (Bernhardsson et al. 2013). These genetic associations enable us to use the DGCs to describe the evolutionary history of defence traits. We study the architecture of the bipartite networks in this system, first by comparing networks containing all genotypes in each of the two gardens, and then comparing the networks associated with the South versus North DGC in each garden. An approach including both common garden comparisons and the knowledge of the evolutionary history of the aspen collection allows us to discuss the relative contribution of environmental variation and of evolutionary constraints in the architecture of networks.

**Table 1:** Geographic and climatic data for the two SwAsp Collection common gardens. Climatic data mean values are shown for the period 2002–2011. PPFD = photosynthetic photon flux density. Sunshine duration and photosynthetic photon data are extracted from the STRÅNG model (Landelius et al. 2001)

	Garden	
	South	North
Location	Ekebo, Skåne	Sävar, Västerbotten
Latitude	55.943504	63.88868
Longitude	13.10866	20.54549
Elevation (m)	76	9
Annual precipitation (mm)^a^	656	491
Mean annual temperature (°C)^a^	8.88	3.83
Photosynthetic photons, May–September (μm s^−1 ^m^−2^)	937	932
CIE-weighted UV irradiance, May–September (mW m^−2^)	17,645	14,860
Annual sunshine duration (hours)	207	242
No. of days with minimum temperature above 5°C^a^	196	106
Hardwood volume growth on forest land (m^3^ ha^−1^)^b^	840,000	120,000

a Data from nearest weather stations.

b Regional data from the Swedish National Forest Inventory.

Based on the literature mentioned above, we had the following expectations regarding the architecture of networks. (1) The arthropods we study spend most of their life cycle on the aspen, so that the network is based on intimate antagonistic interactions, and we expected the overall networks to be modular. (2) Because of the large differences in energy and tree biomass conditions between the two gardens, we expected consistent effects of environmental variation in the architecture of the networks from the two gardens, regardless of the aspen genotypes and their genetic background. (3) We predicted that evolutionary history will also play an important role and that in both gardens networks that associate with southern genotypes will differ from those that associate with northern genotypes. Indeed, previous work suggests that the two genotype populations differ in traits such as tree growth rate and defensive compounds (Bernhardsson et al. 2013). Such traits affect generalist and specialist herbivores in asymmetric ways (Müller-Schärer et al. 2004; Loeuille and Leibold 2008) and will therefore affect modularity as well as nestedness. While our results partly confirmed these expectations, we also found that environmental constraints play a larger role than evolution in our system.

## Materials and Methods

### Field sites

In 2003, 116 aspen (*Populus tremula* L.) trees were selected from 12 regions in Sweden ranging from south to north (55°N–66°N) and east to west (12°E–22°E). Trees were cloned by root propagation in 2003, and at least, four replicates of each genotype were planted in 2004 at each of two common gardens at the Swedish Forestry Research Institute (Skogforsk), in Ekebo (55.9°N) and Sävar (63.9°N). Hereafter, we refer to Ekebo as the southern garden and Sävar as the northern garden. Trees were planted in a randomized block design with at least one replicate of each genotype per block. The gardens were fenced to exclude mammals and weeded annually. Garden locations and geographic and climatic variables are detailed in Table1.

### Field surveys

The aspen canopy attracts a variety of arthropod herbivores. Many species are specialists on *Populus* species. Surveys of herbivorous arthropods were conducted in 2008 when the trees were 5 years old. The foliage on each tree was surveyed exhaustively for arthropod herbivore morphospecies as detailed by Robinson et al. (2012). Here, we report data collected in the middle of the season for arthropod herbivore activity: 15–16 July in the southern garden and 7–9 July in the northern garden. Arthropod herbivore morphospecies identified on the aspens are listed in Table2. Raw data are available (Robinson et al. 2015).

**Table 2:** Arthropod herbivore morphospecies identified on the aspens. Codes refer to the numbers used in Figures3, 4, and 6

Code	Species
1	*Aceria varia*
2	*Aulagromyza*
3	*Byctiscus betulae*
4	*Byctiscus tremulae*
5	*Caliroa tremulae*
6	*Calymnia trapezia* larva
7	*Cerura vinula* larva
8	*Chrysomela* larva
9	*Contarinia petioli*
10	*Contarinia tremulae*
11	*Cryptocephalus sexpunctatus*
12	*Dasineura populeti*
13	*Eriophyes diversipunctatus*
14	Geometridae larva, green
15	Geometridae larva, green, yellow
16	*Harmandia cavernosa*
17	*Harmandia globuli*
18	*Harmandia tremulae*
19	*Lathoe populi* larva
20	Leaf cluster tier
21	Lepidopteran larva, black
22	*Nyclela* larva
23	*Orgyia antiqua* larva
24	Perpendicular lepidoptean roll
25	*Phoesia tremulae* larva
26	*Phratora vitellinae*
27	*Phyllobius*
28	*Phyllocnistis labyrinthella* larva
29	*Phyllocnistis unipuntella* larva
30	*Phyllocoptes populi*
31	Sawfly larva, brown
32	Sawfly larva, green
33	*Tethea or*
34	Tortricid roll
35	Two leaf roll
36	Waxy aphid
37	Woolly aphid
38	*Zeugophora* larva

### Genotype groups

High heritability for plant functional traits and arthropod community measures have been reported from the SwAsp common gardens, indicating high consistency of phenotypes between replicates of a genotype (Robinson et al. 2012). Given this high clonal repeatability, the replicates of each genotype were pooled to give a data set with a total of 113 surviving genotypes in the southern garden and 111 genotypes in the northern garden. Genotype means were calculated for all plant traits and counts of arthropod morphospecies.

### Plant traits

Data on plant growth (stem height and diameter), morphology (leaf area, specific leaf area, and petiole length), and leaf phenolics (condensed tannins and total phenolics) were collected as detailed in Robinson et al. (2012). Extra-floral nectaries (EFNs) at the junction between the leaf base and petiole were quantified on all genotypes, by calculating the ratio of leaves where nectaries were present to absent, for all leaves on the lowest primary branch of each tree. Bud flush and bud set dates were recorded using the method of Luquez et al. (2008), and growth period calculated as the number of days between bud flush and set. Raw data are available (Robinson et al. 2015).

### Evolutionary history of genotypes

Aspen genotypes in the SwAsp collection have been categorized by a collection of single nucleotide polymorphisms (SNPs) for inducible defence genes. Bernhardsson and Ingvarsson (2012) reported geographical grouping based on SNP composition at these loci and defined predominantly southern, central, and northern clusters of genotypes (termed here Defence Genotype Clusters, DGCs). The origins of the genotypes collected from across Sweden, shaded by DGC, and the location of the two common gardens, are shown in Figure2. We used this DGC classification as an indicator of evolutionary history of the aspen genotypes.

### Network metrics

Ecological network descriptors were calculated for each of the two gardens on the following genotype groups: (1) all genotypes, irrespective of DGC (including genotypes that were not scored for SNPs and those that Bernhardsson et al. (2012) categorized as geographically “central”, that is, neither North nor South DGCs); (2) only genotypes from the southern cluster of genotypes based on mapped defence SNPs (South DGC), and (3) only genotypes from a predominantly northern cluster based on defence SNPs (North DGC). The rationale behind including all genotypes (1) was to maximize statistical power by the inclusion of the 30 genotypes that were either not scored for SNPs or fell into a geographically central DGC. The central DGC comprised only ten genotypes, which we consider insufficient for the calculation of network metrics as an independent DGC.

We calculated network metrics in R (R Core Team, 2013) using the networklevel function of the bipartite package (Dormann et al. 2008) to obtain the number of nodes *N* (plant genotypes, insect species), the number of links *L*, the connectance (the ratio between the number of observed links and the number of links that would be observed if all nodes were connected: *L*/*N*(*N*−1)) and nestedness of the data matrix for each garden and DGC. Default parameters were chosen for nestedness (Dormann et al. 2009) and significance of nestedness compared to 100 null models using the method “vaznull” (Vazquez et al. 2007), which converts the original data matrix to a binary matrix and randomizes the interactions, while maintaining the same marginal totals and connectance identical to the original data matrix (Vazquez et al. 2007). Modularity (Q) was calculated on matrices for each garden and DGC using the ComputeModules function in bipartite, which uses the QuanBioMo algorithm for quantitative data matrices described by Dormann and Strauss (2013). The optimal number of steps taken for the algorithm to attain the final module configuration was tested by increasing the number of steps taken to reach a configuration after which Q did not increase (data not shown), resulting in the consensus number of steps used; in the case of our data matrices, this was 1.5 × 10^7^. Modularity was tested against 100 null models also using the method “vaznull.” This yielded a score, *z*_Q_, equivalent to the *z*-score of a normal distribution (Dormann and Strauss 2014). Following the ComputeModules function, the czvalues function extracted connection values (*c*) and participation values (*z*), indicating the contribution of an aspen genotype or an arthropod morphospecies between and within modules, respectively (Dormann and Strauss 2013).

Paired difference index (PDI, Poisot et al. 2012) was calculated for the aspen genotypes using the specieslevel function of the bipartite package in R. Here, we interpret the inverse of PDI (1-PDI) as a measure of the degree of vulnerability of a genotype to arthropod herbivores, where vulnerability = 0 indicates the lowest attractiveness (such that the plant genotype hosts one herbivore species) and vulnerability = 1 indicates maximum vulnerability (the plant hosts all species). The PDI distributions were compared using two-tailed Komolgorov–Smirnov tests in R. One-way analysis of variance (ANOVA) was conducted in R to test for variance in PDI (dependent variable) explained by module group (independent variable) in each data matrix.

### Partial least squares projection to latent structures – discriminant analysis (PLS-DA)

Trait-matching was conducted using PLS-DA analysis as a means to identify phenotypic traits best explaining the module membership of aspen genotypes. PLS uses a dimension reduction approach to configure the data matrix into few dimensions followed by model testing on a leave-one-out basis, resulting in *R*^2^ scores indicating the explained variance of the model and *Q*^2^ indicating the predictive variance of fit (described by Eriksson et al. 1995; Wold et al. 1989). In addition to its use as a predictive tool to assign groups to a data set, the method can be used to extract scores for each predictor variable indicating its importance in the PLS-DA projection, thus its importance in explaining the response variable. Therefore, we used variable importance on projection (VIP) scores to indicate the traits of highest importance in explaining module membership. PLS-DA is a multivariate method that, unlike parametric methods, tolerates data sets with highly correlated variables (Eriksson et al. 1995). The SwAsp phenotype data include many highly correlated phenotypes owing to strong latitudinal gradients in growth-related traits such as bud set and growing season length, and larger trees supporting the highest abundance of herbivores (Luquez et al. 2008; Robinson et al. 2012; Bernhardsson et al. 2013). For each data matrix, data were scaled and mean-centered. Module class of the aspen genotype was set as the y-variable (factor) and aspen phenotypic traits as the x-variables (explanatory variables), and the discriminatory power of the x-variables to separate the module groups was tested using the plsDA function in the package DiscriMiner in R (Sanchez 2013). The VIP scores generated by the plsDA function were used to indicate the explanatory variables (phenotypes) explaining the most variation in the module class. Phenotypic traits with higher VIP values (>1) were considered most important in explaining module membership (Chong and Jun 2005).

### Figure construction

Bipartite graphs and interaction matrices were produced using the plotweb, visweb, and plotModuleWeb functions of the bipartite package in R (Dormann et al. 2008). PLS-DA loading plots were constructed using the plsDA function of the DiscriMiner package in R (Sanchez 2013).

## Results

### Environmental and evolutionary effects on network architecture

**Figure 3:**
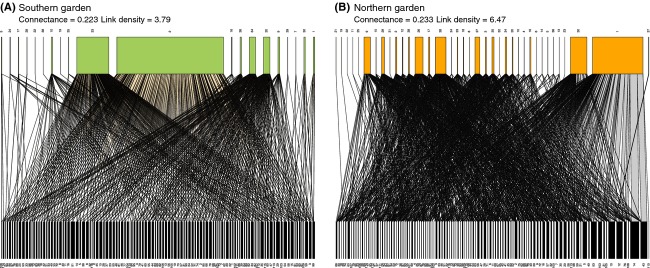
Bipartite graphs for all genotypes in (A) Southern garden and (B) Northern garden. The upper and lower boxes represent the higher trophic (arthropod morphospecies, detailed in Table2) and lower trophic (aspen genotypes, each assigned a unique number) levels, respectively. Lines connecting upper and lower boxes represent interactions between morphospecies and aspen genotypes, and line thickness is scaled to the number of interactions.

The two common gardens experience contrasting environmental conditions (Table1) with the most notable differences, growing degree days (temperatures above 5°C), and hardwood productivity on forest land, resulting from latitudinal distance and soil resource availability, which in turn influence growth potential. Median tree height in the southern and northern gardens was 218 and 134 cm, respectively. These differences in tree size did not negatively influence the number of interactions in the bipartite networks; in the northern garden, the number of links per species was greater than the southern garden for all genotype groups (Table3). Bipartite graphs indicate that web structure differed between the two gardens (Fig.3) with two notably dominant arthropod morphospecies and no clearly preferred or rejected aspen genotypes in the southern garden, compared to an even more complex network with more links in the northern garden. Connectance values, however, were similar between gardens and for all genotype groups (Table3).

**Table 3:** Community network metrics for the south and north SwAsp common gardens, showing values for all aspen genotypes, and the subsets of genotypes: South defence genotype cluster (South DGC) and North defence genotype cluster (North DGC)

Garden	Genotypes	No. Genotypes	No. Herbivores	Link density	Connectance	Nestedness (100-Temperature)	Nestedness *P*-value	No. Modules	Modularity *Q*	*z*_Q_
South	All	20	113	3.79	0.223	85.03 n.s.	0.308	5	0.156***	46.07
	South DGC	17	52	3.55	0.277	81.95 n.s.	0.069	4	0.139***	71.91
	North DGC	17	31	2.77	0.252	87.65 n.s.	0.094	6	0.186***	22.40
North	All	37	111	6.47	0.233	82.64***	<0.001	3	0.319***	51.68
	South DGC	34	52	6.09	0.296	75.01***	<0.001	4	0.289***	54.71
	North DGC	25	32	3.49	0.249	79.18***	<0.001	5	0.444***	47.78

Calculations are based on quantitative data on tree genotype means. Nestedness is expressed as 100-temperature, such that higher values indicate higher nestedness (0 = cold; 100 = perfect nestedness), and significance indicated as n.s. (nonsignificant) or

*** (*P* < 0.0001). *z*_Q_ is the null model comparison with modularity *Q* scores.

**Figure 4:**
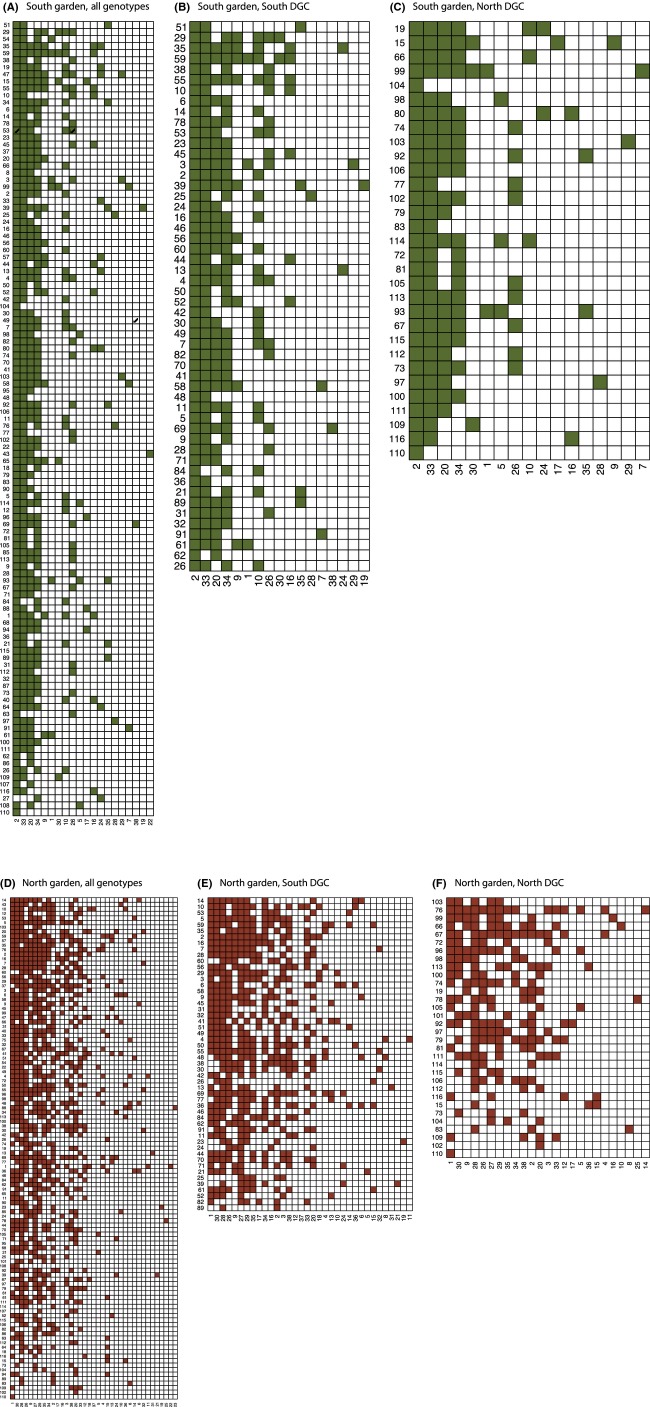
Interaction matrices depicting nestedness in all genotype groups in both gardens. Arthropod morphospecies (*x*-axis) are numbered using the codes detailed in Table2. A shaded square in the matrix indicates an interaction between an aspen genotype (each aspen genotype has a unique number, *y*-axis) and a morphospecies (*x*-axis, detailed in Table2).

Nestedness structure was apparent in both gardens and all genotype groups, as depicted by interaction matrices (Figs.4), although only significant in the northern garden (Table3). For each web (All, South DGC and North DGC), values of nestedness were higher in the Southern Garden compared to the Northern Garden, although nestedness was only significant in the northern garden. The North DGC exhibited higher nestedness than the South DGC in both gardens (Table3).

### Environment, not evolutionary history, shapes plant vulnerability

**Figure 5:**
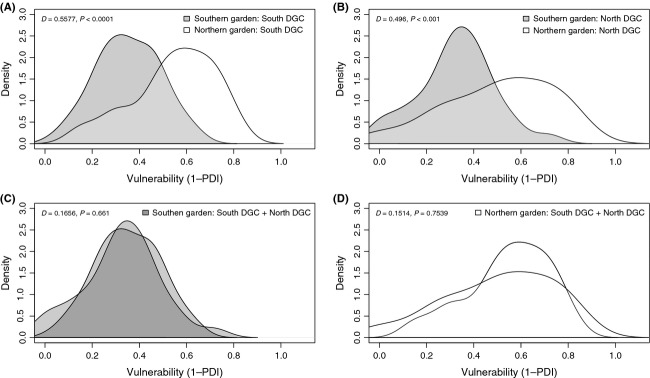
Plant vulnerability, as estimated by (1-plant PDI), in all genotype groups in both gardens. High vulnerability indicates attractive (or less resistant) aspen genotypes due to interactions existing with many herbivores. The distribution density of vulnerability is compared for environmental and evolutionary effects: (A) South DGC in both gardens and (B) North DGC in both gardens indicate environmental effects of the common garden, while (C) North and South DGCs in the south garden and (D) North and South DGCs in the north garden estimate the influence of DGC (evolutionary history) within each garden. The Komolgorov–Smirnov D statistic, testing for differences in vulnerability distributions, is stated for each comparison.

Comparisons of the vulnerability distribution of the South DGC between gardens (Fig.5A) and the North DGC between gardens (Fig.5B) showed significantly higher values in the northern garden, indicating a higher frequency of vulnerable genotypes and less specialization in the northern garden. By contrast, the distributions of vulnerability for the two DGCs within the southern garden (Fig.5C) and northern garden (Fig.5D) did not differ. The median arthropod PDI in the southern garden was 0.31, compared to arthropod PDI of 0.23 in the northern garden, indicating that the degree of herbivore generalization is greater in the northern garden. Overall, there was a clear effect of the environment (garden) and absence of effect of evolutionary history (DGC) on arthropod specialization and aspen vulnerability using the PDI metric.

### All networks exhibit modular structures

**Figure 6:**
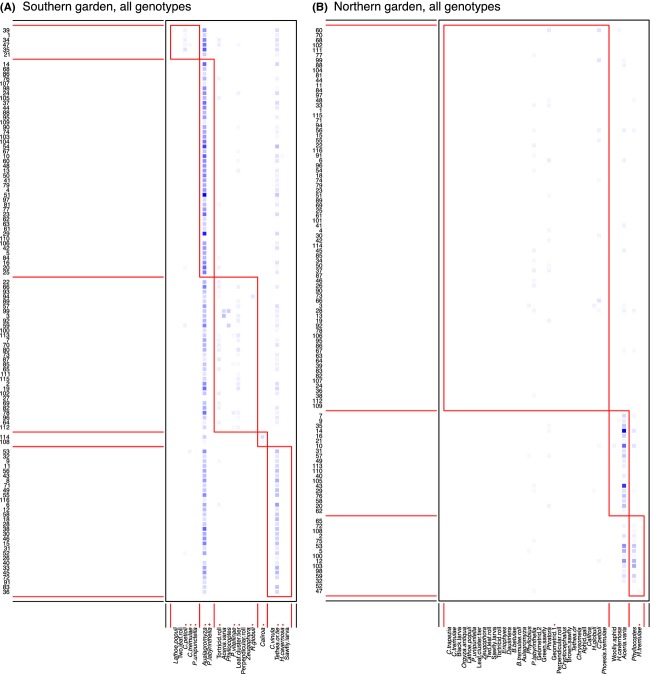
Interaction matrices indicating modularity in all genotype groups in both gardens. Arthropod morphospecies (*x*-axis) are numbered using the codes detailed in Table2. A shaded square in the matrix indicates an interaction between an aspen genotype (each aspen genotype has a unique number, *y*-axis) and a morphospecies. Darker colors of the shaded squares indicate more frequent interactions. Module groups assigned by the QuanBiMo algorithm run with 1.5 × 10^7^ steps are outlined in red. Modularity (*Q*) is stated for each interaction matrix. A ‘+’ indicates morphospecies with significant connectivity between modules (c values). Asterisks denote morphospecies with significant z scores (coefficient of within-module participation).

Modularity (*Q*) values calculated on all data matrices (all genotype groups in both gardens) were notably highly significant in all cases (Table3), with *z*_*Q*_ values in excess of the threshold for significance in the *z*-distribution (i.e., in larger than 2: Dormann and Strauss 2014). Overall, the *Q* values were higher in the northern than southern garden, and within each garden, the highest *Q* values were observed in the North DGC and the lowest in the South DGC. The matrix of all genotypes showed intermediate *Q* values, as expected from the inclusion in the same matrix of both DGCs. These observations suggest an effect of both environmental and evolutionary influences on modularity. Interaction matrices for the two gardens indicate the module configuration (Fig.6) in which herbivore morphospecies with significant connection values, *c*-scores above 0.65 (Dormann and Strauss 2014), are asterisked indicating distribution across modules. None of the herbivores demonstrated consistent contribution to module configuration across the different networks (data not shown). One leaf-mining microlepidopteran (code 28, *Phyllocnistis labyrinthella)* showed a participation *z*-score of 2.58, marginally above the significance threshold of 2.5 (Dormann and Strauss 2014).

### Mechanisms underlying modular structure

**Figure 7:**
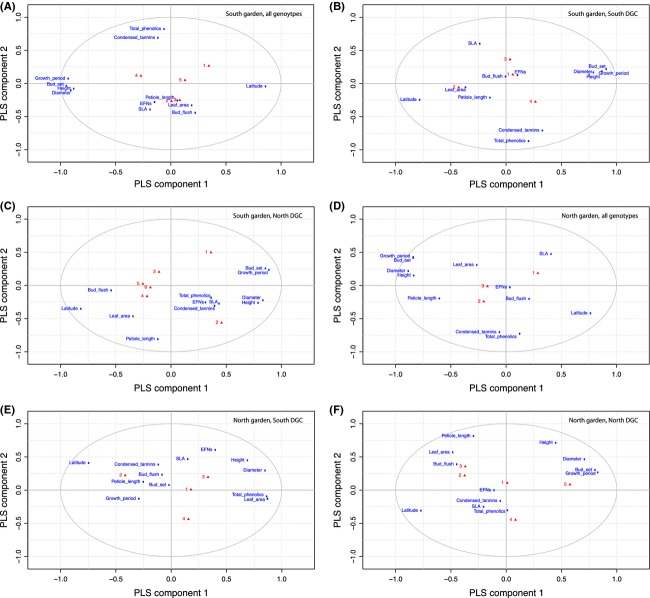
PLS-DA loading plots for all genotype groups in both gardens. In all data sets, the first two components are used in the model. Labeled blue squares indicate trait loadings. Red triangles are the loaded *y*-values (module number).

**Figure 8:**
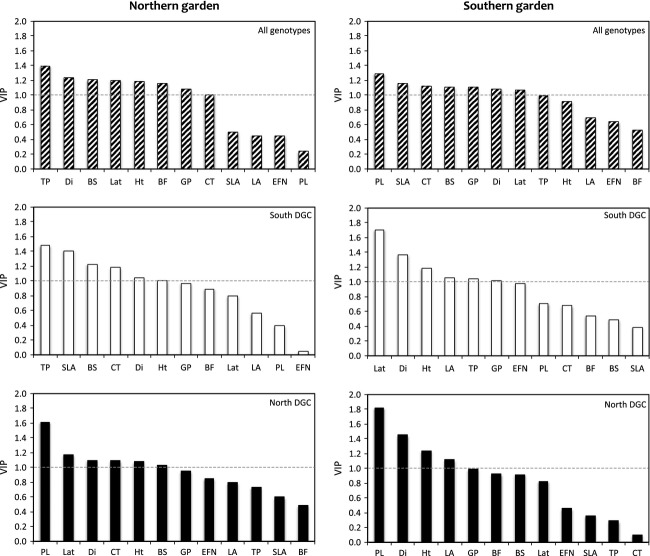
Ranking of VIP (variable importance on the projection) scores to indicate the phenotypic traits most important in explaining module membership in each data matrix (garden and DGC). Traits are ranked from left (highest VIP) to right (lowest VIP). The effects of the north garden (left column) and south garden (right column) and are illustrated in all genotypes, (top), the south DGC (center) and the north DGC (bottom). A dashed line indicates the VIP threshold of one, above which traits are considered significantly important to explain the model. Trait abbreviations: Lat = tree latitude at origin; Ht = tree height; Di = stem diameter; BF = date of bud flush; BS = date of bud set; GP = growing period length; LA = individual leaf area; SLA = specific leaf area; P = petiole length; EFNs = extra-floral nectaries; CT = condensed tannins; TP = total phenolics.

Environmental as well as evolutionary history can lead to variation in traits that determine the number and configuration of modules in a network and may therefore explain the systematic variations in modularity described above. Trait-matching is an approach that explores whether plant phenotypic traits might be causative of module structure. We employed PLS-DA to analyze the variables (phenotypes) with the highest projection on the PLS model of each data matrix. The PLS-DA model fit (*R*^2^) and predictiveness (*Q*^2^) values are shown in Table3. The PLS-DA loading plots (Fig.7) indicate the proximity of modules (each assigned a number) to the loading values of specific plant traits in the PLS projection. We plotted the VIP scores for all phenotypic traits to test for variation explained in all module groups in the southern and northern garden (Fig.8). In all data matrices, several phenotypes correlated with growth and biomass accumulation (height, diameter, latitude, bud set date, and length of the growing season) contributed significantly module membership (VIP > 1). In the northern, but not southern, DGC, petiole length ranked as an important variable. In the southern DGC, only one defensive trait, total phenolics, consistently ranked as an important phenotype. The production of extra-floral nectaries, a leaf trait providing nectar rewards to ants implicit in defence against herbivores, did not contribute as an influential trait in either garden.

**Figure 9:**
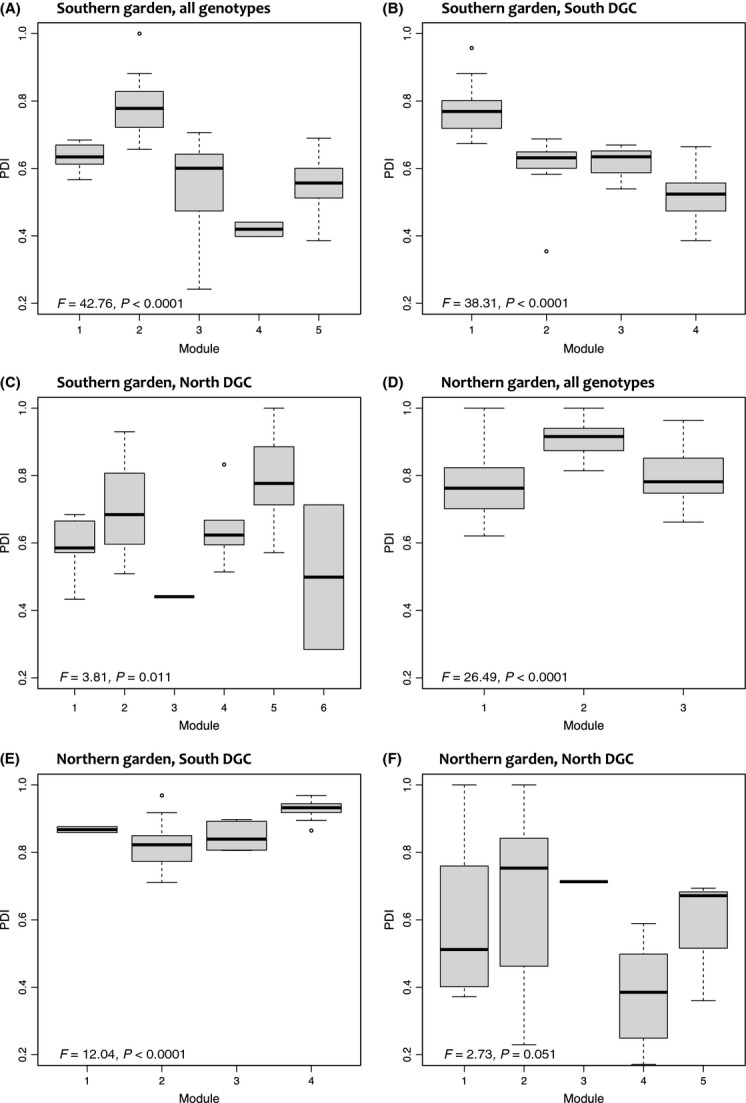
Boxplots to illustrate the matching of module groups (module number) with PDI values of the constitutive genotypes, tested using one-way ANOVA with module as the response variable. Boxplots are shown (A–E) for each genotype group and DGC.

We further tested whether plant vulnerability (1–PDI) could influence modularity, using ANOVA. PDI explained a significant amount of variance in module class in both the southern and northern gardens (*P* values between 0.051 and <0.0001, Fig.9), revealing an effect of plant vulnerability on module structure.

## Discussion

We present the first results of intraspecific network metrics based on a collection of aspen genotypes in two common gardens differing in productivity and their interactions with arthropod herbivores. Antagonistic networks are most often modular (Krause et al. 2003; Thébault and Fontaine 2010). In our case, modularity was consistently significant among networks. The pattern of modularity *Q* values was the same in both gardens: highest and lowest *Q* values in the north and south DGCs, respectively, with intermediate values when all genotypes were considered together. These patterns of modularity could be expected due to the interaction intimacy that exists between aspen and its herbivores: Most of the arthropods in our study spend a large part of their life cycle on the aspen, making for an intimate interaction with hosts (Ollerton 2005). Previous studies have suggested that such intimate antagonistic networks were all the more likely to be modular (Guimaräes et al. 2007; Van Veen et al. 2008), although the intimacy effect on modularity or nestedness does not seem consistent in antagonistic webs when compiling 95 different networks (Fontaine et al. 2011). Nestedness was generally lower in the northern garden; however, strong statistical evidence was lacking for the higher patterns of nestedness in the southern garden. Nested architecture commonly arises in networks (Ings et al. 2009). Some have suggested that nestedness can actually be seen as “null model” structure because, if uneven density distributions exist within each of the two groups making the bipartite network, then nested structures may arise from the random encounters of individuals (Lewinsohn and Prado 2006; Krishna et al. 2008). Interestingly, we do get nested structures for all webs in the northern garden, but nestedness in our case cannot be explained by differences in abundances. Indeed, in our common gardens, clones are planted with roughly equal frequencies (see the even distribution of abundances on the aspen side in Figs.4). Instead, our results suggest that nestedness depends on the environmental variation existing between the two gardens and that a hierarchical organization of bipartite connections is more likely to occur in the more productive southern garden, although the networks overall appear to be more loosely defined under the same conditions. The less distinct hierarchy in the northern garden could potentially be a result of enhanced concentrations of defence compounds in the harsher north (Visnawath et al. 2012). This would likely lead to more distinct phytochemical properties of the expressed tree phenotypes which might constrain and define the bipartite interactions into a network that expresses a higher degree of modularity.

Effects of the environment are dominant in determining the network architecture. The total network in both gardens is always modular, and nestedness is consistently significant in the northern garden, regardless of the network (total, North DGC, and South DGC). Several explanations may be proposed. First, it is possible that variation in architecture is determined by abiotic factors. Particularly, the two gardens have contrasting conditions in terms of energy (available nutrients and solar energy influx) and in terms of climate (mean temperature and precipitation). Based on historical records of Quarternary climate change, climatic conditions have been shown to influence the nestedness and modularity of mutualistic networks (Dalsgaard et al. 2013), annual precipitation, and temperature variations being particularly important factors. Changes in the abiotic environment can have direct impacts on the regional herbivore species pool through local species sorting, or by modifying the expression of traits (phenotypic plasticity). In either case, such abiotic factors constrain the trait-matching or trait-avoidance processes that affect aspen–herbivore interactions in our system (Bernhardsson et al. 2013). We acknowledge, however, that the structural differences between the two gardens cannot be directly linked, for lack of replication, to any single differences in abiotic environment listed in Table1. Similarly, we cannot exclude the role of other differences (e.g., regional community composition or habitat structure). However, our use of common gardens, combined with our knowledge of the evolutionary history of aspen genotypes, allows us to assess that such location differences are more important for the structure of the network, compared to the aspen genetic composition we chose to build the network. In the context of current global change, this primacy of environmental constraints in determining the structure of ecological webs leads to interesting questions. Ecological web structure affects the resilience of natural communities (Thébault and Fontaine 2010) and their invasibility by new species (for instance, coming from southern latitudes) (Romanuk et al. 2009). In this light, our empirical results are in agreement with theoretical studies that suggest that species interactions and network structures will constrain the fate of natural communities facing global changes (Lavergne et al. 2010; Norberg et al. 2012).

Within each garden, networks based on the North DGC had consistently higher nestedness and modularity when compared to networks based on the South DGC. This suggests a role of the evolutionary history of plants in driving the architecture of the network. This role of evolution is, however, weaker compared to the environmental signal, nestedness being not significant in the southern garden and significant in the northern garden. We know of only one other study (Thompson et al. 2013) similarly studying the interplay and hierarchy of environmental and evolutionary processes in shaping the structure of networks, however, for a very different type of system (marine mutualistic goby–shrimp communities), and using phylogenies to discuss evolutionary components (while we focus on intraspecific phenotypic and genotypic variation). Interestingly, in spite of these differences, the two studies reach the same conclusion: While both environmental variation and evolution matter in the context of network nestedness, environment plays a larger role.

Links between network architecture and evolutionary dynamics have been proposed by different models. Cattin et al. (2004) show how nested diets may emerge from the phylogenetic correlations of diet among consumers. Valdovinos et al. (2012) suggest that nestedness may arise from optimal foraging constraints associated with pollination. These two works are therefore based on adaptation of the consumer guild. In contrast, we use our knowledge of the evolutionary history of the plant group to investigate how evolution may affect nestedness and modularity. This also allows us to investigate which traits of the plants matter for the emergence of nestedness and modularity.

We compiled trait data (detailed in Robinson et al. 2012) potentially affecting pairwise interactions and therefore influencing network structure. The PLS-DA model *R*^2^ values (Table4) indicate that the model represents a substantial amount of variation in the traits examined; however, the low *Q*^2^ values suggest poor predictive power of the model. The PLS-DA loading plots (Fig.7) show clear spatial separation of the groups of phenotypic traits examined; for example, growth-related traits (height, diameter, bud set, growth period, and latitude) influence the first component in the majority of the plots, and associated leaf traits such as condensed tannins and total phenolics also aggregate. This consistency suggests that, despite weak predictive ability of the model, the phenotypic data are of good quality and we infer that the lack of any very strong association of module groups with traits is due to the similar importance of many variables for module formation, in agreement with previous work on the SwAsp collection (Robinson et al. 2012), which found that a number of different traits influence arthropod community composition. One main observation clear from trait-matching is that growth and growth-related traits are important for modularity in both gardens. Growth traits affect the network structure by modifying apparency to herbivores (Feeny 1976), making larger trees more likely to be attacked by many herbivores; however, we found no significant correlations between plant PDI and height (data not shown). In the northern garden, petiole length has substantial influence on module structure; indeed, petiole length is correlated with stem growth in the SwAsp collection and in other *Populus* species (Wu and Stettler 1998; Marron et al. 2007). Where all genotypes are considered, both total phenolics and condensed tannins influence module structure, which fits theoretical predictions that defence compounds decrease the vulnerability of aspens, leading to higher specialization of their herbivores. The proportion of leaves bearing extra-floral nectaries (EFNs), however, did not explain module structure in any division of the data (when either garden or DGC were considered), which is surprising given that that there is moderate-to-high intraspecific variation in this trait and EFNs are implicit in the reward system of ants against arthropod herbivores. To some extent, such trait-matching results can be related to theoretical predictions about network structure. Loeuille and Leibold (2008), for instance, show that a large investment in defences at the expense of growth may induce modularity in the plant–herbivore network, while a more intermediate investment would lead to more connected webs.

**Table 4:** PLS-DA model *R*^2^ and *Q*^2^ values

Garden	Genotypes	*R*^2^Xcum *t*_1_	*R*^2^Xcum *t*_2_	*R*^2^Ycum *t*_1_	*R*^2^Ycum *t*_2_	Global *Q*^2^ *t*_1_	Global *Q*^2^ *t*_2_
South	All	0.354	0.050	0.503	0.086	0.019	−0.035
	South DGC	0.313	0.470	0.065	0.122	−0.011	−0.109
	North DGC	0.376	0.502	0.083	0.189	−0.015	−0.123
North	All	0.344	0.513	0.051	0.082	0.006	−0.059
	South DGC	0.301	0.085	0.410	0.155	0.009	−0.269
	North DGC	0.267	0.123	0.459	0.202	−0.021	−0.237

While we limit our analysis to plant traits, coevolutionary aspects can be equally important for network architecture. As a first approximation, coevolutionary effects can be tested by comparing “local assemblages” versus “nonlocal assemblages”. For instance, in the northern garden, the network associated with the North DGC may be seen as more coevolved as the one associated with South DGC. This kind of comparison in the two gardens, however, does not yield consistent variation in nestedness or modularity in our case (Table3). Furthermore, we currently lack relevant genotypic and phenotypic information on the herbivore side to tackle coevolution in efficient ways. Further investigation is required concerning changes in herbivore diets, related traits, but also on the phylogenetic history of herbivore species. It is also important to develop adapted models to provide more precise predictions regarding how herbivore–plant coevolution may change the architecture of these networks. While coevolution models have been proposed to explain nestedness or modularity, such models most often account either for mutualistic (Bascompte et al. 2006; Nuismer et al. 2012) or host–parasite interactions (McQuaid and Britton 2013).

The networks we consider in the present article differ from most networks on which architecture is studied in several aspects. First, we largely control the plant side of the bipartite network. Aspen genotypes have been planted with homogeneous frequencies, and in a controlled manner, while most studies focus on natural assemblages in which both parts of the network are naturally selected (Jordano et al. 2003; Bascompte et al. 2006; Van Veen et al. 2008; Thébault and Fontaine 2010; Fontaine et al. 2011; Thompson et al. 2013). While to some extent, this limits the comparisons we can make with other works, and it also has very important advantages. For instance, it is generally unclear how network architecture can be explained simply by random encounters of individuals of species having dissimilar frequencies (Lewinsohn and Prado 2006; Krishna et al. 2008; Canard et al. 2012) or by ecological and evolutionary dynamics (Bascompte et al. 2006; Thébault and Fontaine 2010; Fontaine et al. 2011). As we fix abundances and maintain them approximately homogeneously on the plant side, we can actually exclude the first type of explanation in our system. Furthermore, the control inherent to common garden experiments allows us to discuss the relative impacts of environmental factors and evolutionary history, an aspect that cannot easily be otherwise discussed. A further aspect by which our study differs from most previous studies concerns the organizational scale we chose for the network. In most other works, nodes of the bipartite networks are species or functional groups (Fontaine et al. 2011). Here, however, plants are all of the same species (aspen), and nodes are made of different phenotypes, making for an “intraspecific” network. This may be justified by the importance of the genotypic variation of trees in structuring their surrounding communities (“community genetics”: Whitham et al. 2003). Such observations suggest that, at least in large, dominant species such as trees, genotypes can be relevant functional entities, here serving as the basis for the definition of network. This also raises interesting issues. First, for a given system, what is the relevant organizational scale to tackle network structures? Second, do networks exhibit similar structures across different organizational scales? Network ecology increasingly turns to applications in ecosystem services, for instance, regarding the provision of biological control or pollination services (Bohan et al. 2013; Loeuille et al. 2013). In an agricultural context, management usually selects for traits or genotypes of crops, so that an assessment of network functioning at this organizational scale is urgently needed.
